# Examining Racial and Ethnic Disparities in Nurse Burnout, Intent to Leave, and Student Debt Burden: A Cross-Sectional Study

**DOI:** 10.1097/AJN.0000000000000003

**Published:** 2025-09-25

**Authors:** Jacqueline Christianson, Kyla Aiyeku, Abiola Keller

**Affiliations:** **Jacqueline Christianson** is an assistant professor and **Abiola Keller** is an associate professor at the Marquette University College of Nursing, Milwaukee, WI, where **Kyla Aiyeku** is a graduate nursing student. Contact author: Jacqueline Christianson, jacqueline.christianson@marquette.edu. The authors have disclosed no potential conflicts of interest, financial or otherwise.

**Keywords:** burnout, discrimination, diversity, intent to leave the profession, nursing workforce, racism, student debt burden

## Abstract

**Background:**

A racially and ethnically diverse nursing workforce can only enhance the profession's ability to serve the widely diverse U.S. population. Embracing differences allows nurses to develop a broader understanding of each other and of their patients. Yet many nurses of color face unique challenges that can adversely impact their well-being and their longevity in this profession.

**Purpose:**

The purpose of this descriptive cross-sectional study was to examine levels of burnout, intent to leave the profession, and student debt burden among nurses who self-identified as Asian, Black or African American, or Hispanic, and compare these to the levels reported by their White counterparts.

**Methods:**

A survey of U.S. hospital nurses took place from January through March 2023, measuring levels of burnout, intent to leave the profession, and student debt burden. Differences regarding self-identified race and ethnicity were then examined using regression analyses.

**Results:**

Asian participants had significantly higher burnout scores and were more likely to report intent to leave the profession than their White counterparts. Hispanic participants were also more likely to report intent to leave. Black and African American participants reported significantly higher student debt burdens than participants from other groups.

**Conclusions:**

This study's findings attest to racial and ethnic disparities regarding nurse burnout, intent to leave the profession, and student debt burden. They raise concerns about the long-term retention of Asian and Hispanic nurses and about barriers to entering the profession among Black and African American nurses. Failure to effectively address racial and ethnic disparities in the workplace jeopardizes workforce diversity. It's our hope that the study findings will spark a larger conversation about how the experiences of nurses of color differ from those of their White counterparts and how disparities can be effectively redressed.

The nursing profession can only benefit from having a diverse workforce, one truly capable of serving various populations. In recognition of this need, in 2023, the American Nurses Association (ANA) and two sister organizations jointly released the *ANA Enterprise 2023-2025 Strategic Plan*, which prioritizes the advancement of “diversity, equity, inclusion, belonging, and anti-racism to improve nursing practice and work environments.”^1^ To successfully advance these laudable goals, it must be acknowledged that nurses of color—those belonging to racial and ethnic minority groups—often aren't afforded the same opportunities and face more adverse circumstances compared to their White counterparts. For nurses of color, examples of inequity include less availability of mentorship, fewer promotional opportunities in both clinical and academic arenas, and experiences of discrimination in the workplace.^2^-^4^ In this study, we examine three variables that might reflect inequity: nurse burnout, intent to leave the profession, and student debt burden.

## BACKGROUND

The current nursing shortage in the United States has reached a crisis level. According to recent reports, the vacancy rate for hospital nurses is about 9.9%, almost 2% higher than it was before the COVID-19 pandemic.^5^,^6^ The shortage is projected to worsen for reasons that include increased patient acuity in an aging population, higher rates of attrition among practicing nurses during and since the pandemic, and nursing faculty shortages.^7^ Chronic understaffing and consequently greater nursing workloads have been linked to care rationing and poorer patient outcomes.^8^ While some attrition in the nursing workforce is due to nurses reaching retirement age, other potentially preventable factors such as work-related burnout and job dissatisfaction can also influence the intent to leave the profession.^9^ Failure to address these factors will likely exacerbate the nursing shortage and could increase the risk of adverse patient care outcomes.

Furthermore, the U.S. nursing workforce does not fully reflect the diversity of the national population. For one, while women make up 50.5% of the population, they account for 88% of RNs.^10^,^11^ Regarding racial and ethnic identity, 11% of RNs identify as Black or African American, compared to 13.7% of the general population; 19.5% of RNs and 9% of the general population identify as Hispanic or Latinx; and 9% of RNs and 6.4% of the general population identify as Asian.^10^,^11^ There is evidence that, although racial and ethnic diversity among RNs has improved overall, it appears to be decreasing for some groups, including RNs who identify as Hispanic.^10^ The underrepresentation of people of color in the health care workforce has negative consequences for patients with regard to health care access, care delivery, and trust in the professionals who provide their care.^12^,^13^ On the other hand, patient care delivered by a diverse team has been associated with both improved patient outcomes and improved professional skills in areas such as team communication and risk assessment.^14^

This is vital to reducing racial and ethnic health care disparities and cultivating an environment of trust between all patients and their providers. Of note, in 2022, the ANA issued the *ANA Racial Reckoning Statement*, in which the organization publicly acknowledged its past actions resulting in the exclusion of nurses of color and committed itself to “addressing the harms that continue today.”^15^ The statement called for action, pledging to “actively engage in a program of diversity, equity, and inclusion within the association” and “partner [with] and support the National Commission to Address Racism in Nursing as it strives to create antiracist practices and environments.”^15^

Low diversity among RNs has been explored in prior research, with some areas receiving more attention than others. Barriers to recruiting, matriculating, and graduating nurses of color have been described in the literature. Disproportionate attrition of prospective nursing students of color has been identified as one contributing factor.^16^ Furthermore, a lack of privilege or social advantage stemming from factors generally outside an individual's control (such as socioeconomic status) is believed to contribute to low nursing education enrollment and retention among racial and ethnic minorities.^17^,^18^ The cost of nursing education also constitutes a barrier that disproportionately affects prospective nurses of color, many of whom report having little information about and poor access to financial support.^13^,^19^ One study, conducted among university students in a prenursing program, found that students from underrepresented minority groups were more likely to require remedial education (thus delaying graduation and increasing costs) and to leave before completing the program.^16^

Regarding practicing nurses, a few studies have examined workplace burnout and intent to leave the profession. Burnout, a syndrome characterized by emotional exhaustion, depersonalization, and disengagement from work, has been associated with premature (prior to retirement) intent to leave the job, the profession, or both.^20^ According to Leiter and Maslach's Areas of Worklife theory, burnout can result from “chronic mismatches” between people and some of or all six dimensions of their workplace: workload, control, rewards, community, fairness, and values.^21^ Accordingly, it stands to reason that a workplace culture of inequity (unfairness) could disproportionately affect nurses of color and increase their risk of burnout. Examples of such inequities abound. For one, research indicates that compared to their White counterparts, nurses of color are less likely to be promoted into leadership positions or high-level academic roles.^22^ Inclusion in the workplace community can also be challenging for nurses of color, given that many have experienced limited access to mentorship and frequent experiences of discrimination in the practice setting.^2^-^4^ Yet it remains unclear whether and how such experiences affect burnout and intent to leave the profession among nurses of color.

To further complicate matters, some nurses have reported the considerable cost of their nursing education—and the resulting student debt—as “anchoring” them to the profession despite a desire to leave.^23^ In light of the financial inequities and challenges already mentioned, this suggests that student debt burden warrants more investigation as a potential factor in intent to leave among nurses of color.

**Study aim**. The purpose of this descriptive study was to examine levels of nurse burnout, intent to leave the profession, and student debt burden among nurses who self-identified as Asian, Black or African American, or Hispanic, and compare these to the levels reported by their White counterparts.

## METHODS

**Design, sample, and setting**. This study was a cross-sectional descriptive survey conducted among RNs, LPNs, and advanced practice RNs (APRNs). The inclusion criteria were being a nurse in current practice and working in an acute care hospital in the United States. Potential participants were selected from state licensure registries in four states—Florida, Oregon, Rhode Island, and Wisconsin—via a computerized random number generator and were recruited by email; a small number were also recruited via the social media platform Facebook. The four states were chosen to promote sample diversity with regard to geographic location, union participation, and prevalence of travel nursing. The recruitment emails and social media posts described the study and provided a survey link. The first survey item was a form asking respondents to provide informed consent; only those who did were given access to the rest of the survey. Approval for the study was obtained from the university's institutional review board prior to data collection.

**Data collection and instruments**. Data were collected from January 16 through March 31, 2023, via Qualtrics. Demographic data of interest included participant gender, highest nursing degree, practice type (such as direct care, leadership, and management), and nursing role (RN, LPN, APRN). Race and ethnicity were assessed using multiple response options from which participants could select all that applied: Asian, Black/African American, Caucasian/White, Hispanic, Native American/Alaskan Native, Native Hawaiian/Other Pacific Islander, and Other. Number of hours worked weekly in all nursing jobs and number of years in nursing were measured to evaluate comparability between groups and were self-reported using a slider bar (options ranged from 0 to 80 and 0 to 60, respectively).

Burnout was assessed using part of the Copenhagen Burnout Inventory, specifically the work-related burnout scale.^24^ This seven-item measure asks participants to answer questions such as, “Are you exhausted in the morning at the thought of another day at work?” Participants respond using a Likert-type scale with five options scored as 100, 75, 50, 25, or 0 points. Depending on the question, response options range from “always” (100 points) to “never/almost never” (0 points) or from “to a very high degree” (100 points) to “to a very low degree” (0 points). The total score is calculated as the mean of the scores for the seven items. A total score of 100 indicates a very high level of burnout. The Copenhagen Burnout Inventory work-related burnout scale has been validated for use among U.S. nurses and has demonstrated strong internal consistency (Cronbach α = 0.87).^24^

Intent to leave the profession was assessed using a binary yes-or-no question: “Do you intend to change professions in the next 12 months?”

Student debt burden was assessed by asking participants to self-report their current outstanding student debt in thousand-dollar units. Responses were collected using a slide bar, with options ranging from 0 to 200.

**Data analysis**. Descriptive statistics (number or frequency, percentages, means, and standard deviations) were employed to describe the sample. Regression analyses were performed to compare differences in means between each racial and ethnic group and with the reference group (White). Racial and ethnic groups were coded as binary dummy variables (that is, a variable was created for each racial and ethnic group, and all participants were coded as yes or no within each group). Because of small sample sizes, three groups (Native American or Alaskan Native, Native Hawaiian or Other Pacific Islander, and Other) were excluded from the analyses.

Two separate linear regressions were performed to evaluate the relationships between race and ethnicity (independent variable) and burnout score and student debt amount (dependent variables). A binary regression was used to evaluate the relationships between race and ethnicity (independent variable) and intent to leave (dependent variable).

## RESULTS

**Sample**. A total of 691 participants were included in the final sample. The majority identified as White (86.7%) and as female (85.4%). Participants' mean age was 45.2 years, with a range of 24 to 74 years. Most were RNs (89.4%) and worked in direct patient care (80.6%). A post hoc sensitivity power analysis was performed using G∗Power, version 3.1.9.6, which showed that effect sizes of 0.31 or greater were detectable by regression analyses. See Table 1 for more detailed participant characteristics.

**Table 1.** Participant Characteristics (N = 691)

		Among Participants Who Identify as . . . a			
Characteristic	n (%)	Asian (n = 21) n (%)	Black/African American (n = 33) n (%)	Hispanic (n = 41) n (%)	White (n = 599) n (%)
Gender					
Female	590 (85.4)	14 (66.7)	30 (90.9)	32 (78)	514 (85.8)
Male	94 (13.6)	3 (14.3)	2 (6.1)	9 (22)	80 (13.4)
Nonbinary	5 (0.7)	0 (0)	0 (0)	0 (0)	5 (0.8)
Other	1 (0.1)	1 (4.8)	0 (0)	0 (0)	0 (0)
Missing responses	1 (0.1)	3 (14.3)	1 (3)	0 (0)	0 (0)
Highest nursing degree					
LPN	7 (1)	0 (0)	2 (6.1)	0 (0)	5 (0.8)
Nursing diploma (RN)	12 (1.7)	0 (0)	0 (0)	0 (0)	12 (2)
Associate degree (RN)	136 (19.7)	3 (14.3)	4 (12.1)	8 (19.5)	121 (20.2)
Bachelor's degree (RN)	369 (53.4)	11 (52.4)	18 (54.5)	22 (53.7)	318 (53.1)
Master's degree (MSN or APRN)	114 (16.5)	4 (19)	5 (15.2)	5 (12.2)	100 (16.7)
Doctorate (DNP or PhD)	35 (5.1)	0 (0)	2 (6.1)	4 (9.8)	29 (4.8)
Missing responses	18 (2.6)	3 (14.3)	2 (6.1)	2 (4.9)	14 (2.3)
Practice type					
Direct patient care	557 (80.6)	14 (66.7)	27 (81.8)	35 (85.4)	481 (80.3)
Management/leadership	70 (10.1)	2 (9.5)	3 (9.1)	2 (4.9)	63 (10.5)
Informatics	4 (0.6)	0 (0)	0 (0)	0 (0)	4 (0.7)
Education	10 (1.4)	0 (0)	1 (3)	0 (0)	9 (1.5)
Case management	35 (5.1)	2 (9.5)	0 (0)	3 (7.3)	30 (5)
Other	11 (1.6)	0 (0)	1 (3)	1 (2.4)	9 (1.5)
Missing responses	4 (0.6)	3 (14.3)	1 (3)	0 (0)	3 (0.5)
Nursing role					
LPN	7 (1)	0 (0)	1 (3)	0 (0)	6 (1)
RN	618 (89.4)	18 (85.7)	27 (81.8)	38 (92.7)	535 (89.3)
APRN	59 (8.5)	0 (0)	2 (6.1)	3 (7.3)	54 (9)
Missing responses	7 (1)	3 (14.3)	3 (9.1)	0 (0)	4 (0.7)
Age in years, mean (SD)	45.2 (13.4)	44.3 (11.6)	44.3 (10.3)	39.3 (10.9)	43.1 (10.3)
Nursing hours worked per week, mean (SD)	40.7 (12.2)	42.1 (15.9)	45.9 (17.3)	39.7 (11.3)	45.9 (17.3)
Years in nursing, mean (SD)	17.6 (12.8)	17.7 (12.7)	12.8 (9.4)	12.5 (9.5)	18.2 (13.1)

APRN = advanced practice RN.

a Because participants could select more than one response for race and ethnicity, the number of participants in the four racial and ethnic groups sums to more than 691.

Note: Because of rounding, some percentages may not sum to 100%.

**Burnout**. The mean burnout scores for Black and African American participants (67.8) and Hispanic participants (66.7) did not significantly differ from that for White participants (62.6). The mean burnout score for Asian participants (75.7) was significantly higher than that for White participants *(P =* 0.003).

**Intent to leave the profession**. The percentage of participants indicating an intent to leave nursing was significantly higher for both Asian participants (38.1%; *P =* 0.048) and Hispanic participants (35%; *P =* 0.03), compared to their White counterparts (19.9%). There was no significant difference in intent to leave for Black and African American participants (30.3%; *P* = 0.15) compared to their White counterparts.

**Student debt burden**. Participants who identified as Black or African American reported significantly higher student debt burdens (mean, 70.6 [in thousand-dollar units]; *P* < 0.001) than White participants (mean, 27.5). Participants identifying as Asian (mean, 31.1) or Hispanic (mean, 37.8) did not report significantly different student debt burdens compared to their White counterparts. See Table 2 for detailed regression analyses results.

**Table 2.** Regression Analyses

Dependent Variables				
Work-Related Burnout^a^ (n = 694)	Mean (SD)	β	95% CI	*F* (*df*)	*P*
Racial and ethnic groups:				3.86 (3)	0.009
Asian (n = 21)	75.7 (19.4)	12.93	4.35 to 21.51		0.003
Black/African American (n = 33)	67.8 (19.4)	5.03	−1.87 to 11.94		0.15
Hispanic (n = 41)	66.7 (19.2)	3.97	−2.26 to 10.21		0.21
White^b^ (n = 599)	62.6 (19.6)					
Intent to leave the profession^c^ (n = 687)	n (%)	β	95% CI	χ^2^ (*df*)	*P*
Racial and ethnic groups:				8.83 (3)	0.03
Asian (n = 21)	8 (38.1)	0.91	1.01 to 6.14		0.048
Black/African American (n = 33)	10 (30.3)	0.56	0.82 to 3.79		0.15
Hispanic (n = 40)	14 (35)	0.74	1.07 to 4.12		0.03
White^b^ (n = 593)	118 (19.9)				
Student debt burden^a^ (n = 691) (in thousand-dollar units)	Mean (SD)	β	95% CI	*F* (*df*)	*P*
Racial and ethnic groups:				11.16 (3)	< 0.001
Asian (n = 21)	31.1 (35.4)	3.81	−16.71 to 24.33		0.72
Black/African American (n = 32)	70.6 (60)	42.33	28.34 to 58.33		< 0.001
Hispanic (n = 41)	37.8 (40.6)	10.57	−3.70 to 24.84		0.15
White^b^ (n = 597)	27.5 (37)				

β = standardized regression coefficient; CI = confidence interval; *df* = degrees of freedom; χ^2^ = chi square.

a Linear regression analysis.

b Reference category.

c Binary regression analysis.

Notes: Because participants could select more than one response for race and ethnicity, n can sum to more than 691. Missing responses were not included in analyses. Because of rounding, some percentages may not sum to 100%.

## DISCUSSION

The study found some differences, based on race and ethnicity, in hospital nurses' reported levels of burnout, intent to leave the profession, and student debt burden. It offers insight into how these experiences differ among nurses from different racial and ethnic backgrounds and highlights opportunities for better supporting and retaining nurses of color.

**Burnout**. Study participants who identified as Asian reported significantly higher burnout levels compared to their White counterparts. One reason for this might be the timing of the study. It took place near the end of the COVID-19 pandemic, a time when the prevalence of anti-Asian racism spiked above prepandemic levels.^25^ According to one study, over 60% of Asian nurses reported enduring increased and frequent job harassment and unfair treatment during the pandemic, and those who experienced such discrimination were twice as likely to report burnout.^26^ But racism in the health care workplace is not a new phenomenon. In a number of studies conducted before the pandemic, Asian nurses reported being subjected to demeaning comments related to their race or ethnicity, acts of discrimination from patients and colleagues, and barriers to career advancement.^27^

Despite abundant evidence that Black or African American and Hispanic nurses face similar experiences of discrimination and barriers to advancement,^2^,^3^,^28^ in our study, these nurses reported burnout levels comparable to those of their White peers. One possible explanation concerns self-selection and sample proportions. There were 599 White participants in the sample, far more than there were Black/African American or Hispanic nurses (33 and 41, respectively). It's possible that, in light of White privilege, White nurses felt more comfortable participating than nurses of color did. Thus, although mean burnout scores for Black or African American nurses and Hispanic nurses were higher than for White nurses, the detectable effect sizes (0.31 or greater) might not accurately reflect differences.

Furthermore, it's important to contextualize our findings considering racial and ethnic discrimination both within and beyond the health care workplace. Combs's Bodies Out of Place theory is useful here.^29^ It describes mundane, everyday discrimination as arising out of the conflict between “trespassing” (the presumption that in certain spaces, being Black is not “allowed”) and having equitable access to those spaces. This is relevant since nurses of color don't stop being of color when they leave work. For Black and African American people, the commonality of experiences of discrimination in everyday life can have the effect of normalizing such experiences in the workplace.^30^ Since the tool used in this study is workplace-specific, it may have limited accuracy for users who have normalized workplace discrimination in this way.

A lack of social support from work colleagues and supervisors and, more broadly, a lack of workplace community have been cited as contributors to burnout.^21^ Moreover, unfair procedures and practices tend to “alienate” individuals from the community and, in doing so, could increase their susceptibility to burnout.^21^ Nurses of color have often experienced both unfairness and exclusion from their workplace community. However, neither the instrument used in this study nor two other frequently used instruments, the Maslach Burnout Inventory and the Oldenburg Burnout Inventory, include items that directly assess perceived fairness or community connectedness in the workplace.^24^,^31^,^32^ Thus, these instruments likely fail to fully detect the effects of everyday discrimination on burnout.

**Intent to leave**. Given their significantly higher burnout scores, it is perhaps unsurprising that a significantly higher percentage of participants who identified as Asian indicated an intent to leave the nursing profession compared to their White counterparts. However, a higher percentage of participants who identified as Hispanic also indicated an intent to leave the profession despite reporting burnout levels comparable to those of White participants. One possible explanation for this is that, as discussed above, the three instruments commonly used to measure burnout are not designed to capture the effects of racism and discrimination on burnout. One study among Black nurses found that workplace racism was a factor in impeding career advancement, and was associated with intent to leave a nursing job.^22^ This suggests that experiences of workplace racism or discrimination and burnout may have separate and distinct effects on intent to leave.

**Student debt burden** was significantly greater for participants who identified as Black or African American compared to White participants. We examined education levels among all four participant groups and found no significant differences. Among the Black and African American participants, the finding of a mean student debt burden of over $70,000 is consistent with findings published by the American Association of Colleges of Nursing (AACN), showing that 54% of Black and African American nursing students carry student debt burdens of $55,000 or greater.^33^ But the AACN also reported that 56% of Hispanic nursing students carry student debt burdens of $55,000 or greater, whereas in our study, the Hispanic participants reported a mean student debt burden of under $40,000. The student debt burden borne by U.S. nurses is concerning regardless of where they stand on their educational or professional journey, but it is particularly concerning for nurses of color, in part because they are less likely to be promoted to more lucrative leadership positions.^22^

Moreover, there is evidence that student debt burden serves to anchor nurses to jobs they might otherwise choose to leave,^23^ although more research in this area is needed. We find it notable that, unlike the other participants of color in our study, those who identified as Black or African American did not differ from their White counterparts regarding intent to leave the profession. Although prior analyses of the study sample, published elsewhere, found no significant relationship between student debt amount and intent to leave,^9^ it's possible that race and ethnicity might moderate that relationship. The prospect that student debt burden has an anchoring effect is troubling. Taken together, the likelihood that nurses of color will accrue disproportionately high student debts and then face race- and ethnicity-related barriers to career advancement make recruiting these nurses exceptionally challenging.

**Suggestions for research**. More research is needed to explore the reasons behind the intent to stay or leave the profession among nurses of color. The underappreciated relationship between experiences of workplace discrimination and nurse burnout also warrants further investigation. Intersectionality—a theoretical framework that considers how multiple social categories such as race, ethnicity, gender, sexual orientation, and socioeconomic status intersect in ways that create unique experiences of privilege and oppression^34^—could prove to be a useful framework in future research.

**Limitations**. Because the study design was descriptive and cross-sectional, the findings are associative and cannot imply causation and are limited to the time in which data were collected. The sampling method allows the possibility of self-selection bias, which also limits the generalizability of the findings. Because the number of participants who identified as Black or African American, Asian, and Hispanic were relatively small compared to those identifying as White, statistical significance detection was limited to medium and large effect sizes. This also prevented accurate analysis of more nuanced concepts, such as whether concern for student debt burden might have influenced intent to stay or leave among participants of color. The self-reported nature of all variables may be a limitation, particularly with regard to student debt burden. Nor did we assess participants' workplace experiences of racism or discrimination.

Lastly, it's important to acknowledge that our use of broad racial and ethnic categories cannot discern differences that exist within groups. For example, a nurse who identifies as Indian may have different experiences than one who identifies as Chinese, even though both would probably have self-identified as Asian per the available categories. Every racial and ethnic group represented in this study contained diverse individuals. It is not our intent to imply that the people in any group are homogeneous or that their experiences of racial and ethnic disparities are the same.

## CONCLUSIONS

Both patients and nursing professionals benefit from a diverse nursing workforce, but recruiting nursing students and nurses of color is insufficient. This study's findings attest to racial and ethnic disparities regarding nurse burnout, intent to leave the profession, and student debt burdens. They raise concerns about the long-term retention of Asian and Hispanic nurses and barriers to entering the profession among Black and African American nurses. Failure to fully address racial and ethnic disparities in the workplace jeopardizes workforce diversity. Failure to recruit and retain a diverse workforce denies patients of color the benefits of optimal care, lowers trust in health care professionals, and can lead to poorer outcomes.^12^,^13^ It's our hope that the findings of this study will spark a larger conversation about how the experiences of nurses of color differ from those of their White counterparts, and how disparities can be effectively redressed.
